# Exploration and Clinical Verification of the Blood Co-Expression Genes of Type 2 Diabetes Mellitus and Mild Cognitive Dysfunction in the Elderly

**DOI:** 10.3390/biomedicines11040993

**Published:** 2023-03-23

**Authors:** Yu Zhang, Shengfeng Deng, Hongfei Zhong, Miao Liu, Jingwen Ding, Rulin Geng, Qiuyun Tu

**Affiliations:** Department of Geriatrics, The Fifth Affiliated Hospital, Sun Yat-sen University, Zhuhai 519000, China

**Keywords:** mild cognitive dysfunction, type 2 diabetes mellitus, bioinformatics analysis, GEO database, blood co-expressed genes, qRT-PCR

## Abstract

With the development of society, the incidence of dementia and type 2 diabetes (T2DM) in the elderly has been increasing. Although the correlation between T2DM and mild cognitive impairment (MCI) has been confirmed in the previous literature, the interaction mechanism remains to be clarified. To explore the co-pathogenic genes in the blood of MCI and T2DM patients, clarify the correlation between T2DM and MCI, achieve the purpose of early disease prediction, and provide new ideas for the prevention and treatment of dementia. We downloaded T2DM and MCI microarray data from GEO databases and identified the differentially expressed genes associated with MCI and T2DM. We obtained co-expressed genes by intersecting differentially expressed genes. Then, we performed GO and KEGG enrichment analysis of co-DEGs. Next, we constructed the PPI network and found the hub genes in the network. By constructing the ROC curve of hub genes, the most valuable genes for diagnosis were obtained. Finally, the correlation between MCI and T2DM was clinically verified by means of a current situation investigation, and the hub gene was verified by qRT-PCR. A total of 214 co-DEGs were selected, 28 co-DEGs were up-regulated, and 90 co-DEGs were down-regulated. Functional enrichment analysis showed that co-DEGs were mainly enriched in metabolic diseases and some signaling pathways. The construction of the PPI network identified the hub genes in MCI and T2DM co-expression genes. We identified nine hub genes of co-DEGs, namely LNX2, BIRC6, ANKRD46, IRS1, TGFB1, APOA1, PSEN1, NPY, and ALDH2. Logistic regression analysis and person correlation analysis showed that T2DM was correlated with MCI, and T2DM increased the risk of cognitive impairment. The qRT-PCR results showed that the expressions of LNX2, BIRC6, ANKRD46, TGFB1, PSEN1, and ALDH2 were consistent with the results of bioinformatic analysis. This study screened the co-expressed genes of MCI and T2DM, which may provide new therapeutic targets for the diagnosis and treatment of diseases.

## 1. Introduction

Mild cognitive impairment (MCI) is an intermediate state between normal aging and dementia and a type of cognitive impairment syndrome [1,2]. With the increasing aging population, there are at least 50 million dementia patients worldwide, with 15 million dementia patients in China alone. In China, the number of MCI patients is between 38 million and 77 million [3], and the incidence of cognitive disorders is on the rise. At present, the auxiliary tests for MCI and dementia include body fluid examination (blood, urine, and cerebrospinal fluid), imaging examination (cranial CT, cranial MRI, and functional imaging), electrophysiological examination (electroencephalogram, and evoked potential), and genetic examination. Most treatments are cause-specific, but there has been no satisfactory progress in inhibiting the transformation of MCI into dementia. Alzheimer’s disease (AD) is the most common form of dementia, rapidly becoming a costly and deadly social burden [4]. The main pathological features of AD are Aβ senile plaques and P-tau neurofibrillary tangles [5]. However, many drug therapies targeting two pathological markers, Aβ and P-tau, are ineffective, which may be because most therapies are started at the stage when cognition has already been significantly changed. Therefore, there is an urgent need to start diagnosis and treatment at the early stage when the pathological changes can be reversed, namely the MCI stage. Therefore, early diagnosis is extremely important. At present, the diagnosis of cognitive impairment mainly employs Aβ, Tau, and neurodegeneration or neuronal injury in cerebrospinal fluid or PET as diagnostic criteria [6]. Due to the high trauma, cerebrospinal fluid leakage, increased operation risk of lumbar disc herniation, high detection cost, and other problems related to these methods, many researchers have shifted their target to peripheral blood samples to seek a less invasive and definitive diagnosis method.

A large cross-sectional study found that dementia and MCI were highly prevalent in the older population and had similar risk factors, such as age, gender, education history, smoking, and underlying diseases (hypertension, hyperlipidemia, diabetes, heart disease, cerebrovascular disease) [3]. Type 2 diabetes mellitus (T2DM) is a common metabolic disorder characterized by hyperglycemia, insulin resistance, and relative insulin deficiency [7]. A meta-analysis [8] indicated that the overall prevalence of T2DM (9.1%) in China has been increasing since the 1970s and increases rapidly with age. T2DM plays an important role in the pathogenesis of MCI. Studies have shown that 25–36% of people with diabetes have mild cognitive impairment (MCI) [9]. In addition to the well-known association between T2DM and peripheral nervous system diseases, diabetes may also cause direct damage to the central nervous system [9]. Furthermore, patients with T2DM generally show worse cognitive performance [10] and a higher risk of dementia [11,12]. Previous studies have suggested that T2DM is not only a risk factor for MCI, but also promotes the transformation of MCI into dementia [13]. It has been reported that T2DM acts on the nervous system through insulin resistance, and it is widely believed that T2DM and MCI share several common abnormal molecular and cellular characteristics, including impaired glucose metabolism, insulin resistance, and increased oxidative stress, which are manifested as persistent hyperinsulinemia and hyperglycemia [14]. Brain insulin resistance seems to be an early and common feature in AD patients, and abnormal glucose metabolism may play a dominant role in the progression of dementia, but the specific mechanisms remain elusive. The human brain uses about 20% of glucose [15] to support synaptic activity [16] and 95% of glucose to produce ATP [17]. Insulin independently regulates most glucose metabolism processes. Insulin resistance acts on different brain areas, such as memory, cognition, and energy metabolism. Insulin resistance significantly increases the risk of sporadic AD [18,19], while T2DM increases the risk by 50% [18]. Previous studies have shown that epigenetic variation interferes with protein folding and triggers endoplasmic reticulum stress [20], thereby affecting tau’s post-translational modification and self-assembly of tau from an A-helix to a B-structure [21]. Increased phosphorylation levels, decreased ubiquitination levels, and decreased methylation levels are some of the changes that can be observed after AD translation [22,23,24]. Abnormal glucose metabolism in peripheral blood can damage the blood–brain barrier and lead to abnormal glucose metabolism and insulin resistance in the brain [25]. Advanced terminal glycation (AGE) and O-glcn acylation, mitochondrial dysfunction, reactive oxygen species (ROS), endoplasmic reticulum stress, and calcium homeostasis may lead to neuroinflammation, neuronal apoptosis, Aβ deposition, tau phosphorylation, and NFTs. The disturbance of glucose homeostasis may play a pathogenic role in the development of an increased risk of cognitive impairment. Therefore, early treatment of MCI in T2DM patients is essential to improve their prognosis and delay the progression to dementia.

In conclusion, we hypothesize that T2DM and MCI have a common pathogenesis, and T2DM patients with MCI are more likely to develop AD. If intervention can be made early in the disease, it could delay the development of AD. Hence, this study used biogenic analysis to explore the gene co-expression of T2MD and MCI, explore their common mechanism at the gene level, design clinical and basic experiments for verification, and provide a new strategy and direction for the treatment of dementia.

## 2. Materials and Methods

### 2.1. Acquisition of MCI-Related and T2DM-Related Gene Expression Data

We searched the Gene Expression Omnibus (GEO) database (https://www.ncbi.nlm.nih.gov/geoprofiles/, accessed on 13 May 2022) for gene expression profile data of patients with MCI and T2DM. The selection criteria of microarray datasets and methods for RNA extraction in the dataset are shown in Supplementary S1. There were 3 MCI patients and 3 healthy controls in GSE18390 and 20 MCI patients and 20 controls in GSE140829. There were 18 T2DM patients and 18 control patients in GSE26168 and 8 T2DM patients and 8 control patients in GSE19532. The sample size of some chips was small, so we performed a combined multi-chip analysis for the same disease. In addition, since individual chips came from different platforms, batch calibration, and sample normalization were performed to eliminate differences. However, considering that the gene we studied was only based on blood samples, and the gene expression in other tissues also plays an important role, we selected GSE5281 (hippocampus) and GSE7014 (skeletal muscle) for verification of our conclusions. We substituted the obtained co-expressed genes into validation datasets to verify whether these co-expressed genes were expressed in tissues. To eliminate the differences between platforms, the verification sets are all from GPL570. These datasets come from a public database and therefore do not require ethical approval. Table 1 lists all the details of these datasets.

### 2.2. Construction of Weighted Gene Co-Expression Network Analysis (WGCNA)

Weighted gene co-expression network analysis (WGCNA) is a common method to construct gene co-expression networks (a detailed description is provided in Supplementary S1). We constructed a co-expression network targeting DEGs using a WGCNA package (version 1.69) in R (version 4.2.1, R Foundation for Statistical Computing, Vienna, Austria). We built a weighted adjacency matrix, constructed from the weighted correlation coefficients, and transformed the adjacency matrix into a matching overlap matrix (TOM). Then, hierarchical clustering was performed to identify the modules and to calculate the feature genes. Finally, we evaluated the correlation between phenotypes (e.g., MCI and control samples) and each module by means of correlation analysis, and identified gene-related modules.

### 2.3. Identification of Differentially Expressed Genes (DEGs)

In this study, we preprocessed the data using the limma software package (version 3.50.1) on the R platform and screened for differentially expressed genes (DEGs) between patients and healthy controls. We used the limma software package on the R platform to perform differential analysis of the MCI and diabetes datasets separately. We defined the statistical difference as *p*-value < 0.05. The logFC > logFoldChange and adj. *p*-value < adjustP were considered as up-regulated genes. The logFC < (-logFoldChange) and adj. *p*-value < adjustP were considered as downregulated genes. Finally, the DEGs of MCI and T2DM were intercrossed (only for genes that were both up- and down-regulated) for co-expressed genes (co-DEGs), and the co-DEGs were visualized using the R platform.

### 2.4. Functional and Pathway Enrichment Analyses

We conducted an enrichment analysis to explore the function of DEGs and their more comprehensive biological information. The ClusterProfiler package (version 4.2.2) in R-platform was used to perform GO enrichment analysis and Kyoto Encyclopedia of Genes and Genomes (KEGG) pathway enrichment. The enrichment analysis results were split into three functional categories: biological processes (BP), cellular components (CC), and molecular function (MF). The barplot map and dotplot map were used to visualize the results. The statistical difference was defined as p.adjust < 0.05.

### 2.5. Protein-Protein Interaction (PPI) Analysis and Identification of Hub Genes

We used the STRING 26 (http://string-db.org, accessed on 17 May 2022) online tool to establish PPI networks of MCI, T2DM, and co-DEGs. We set the interaction score to a maximum confidence score of >0.4 and hid disconnected nodes in the network. However, because co-DEGs have weak associations and a crowd of disconnected nodes, we set a low confidence score of >0.150 for co-DEGs. Ultimately, to better understand the interaction relationship between proteins and the functional interaction between the selection hub genes, we visualized and analyzed the PPI network using the Cytoscape (version 3.9.1) software (http://www.cytoscape.org/, accessed on 17 May 2022) Genes in the PPI network were ranked to screen for the top 10 genes in each algorithm that were identified as hub genes. Finally, the co-expressed hub genes for MCI and T2DM were obtained.

### 2.6. Conduction of Receiver Operating Characteristic (ROC) Curves

We evaluated the accuracy of hub gene prediction and used ROC (a detailed description in Supplementary S1) analysis to distinguish MCI from the normal control, and T2DM from the normal control. The pROC package (version 1.18.0) in R-platform was used to build the ROC curves of the hub genes and to measure the area under the ROC curve and the AUC to compare the diagnostic value of the hub genes.

### 2.7. Prediction of Potential Drugs and Traditional Chinese Medicine for MCI and T2DM

The clue online analysis website (https://clue.io/, accessed on 25 May 2022) can predict drugs through gene targets. Upregulated co-DEGs and downregulated co-DEGs were submitted to the L1000 platform. The score value evaluated the results, and the score values ranged from −100 to 100. The closer the value was to 100, the more similar the gene expression was, and the closer the value was to −100, the more different the gene expression was. Thus, we can predict drugs for MCI and T2DM.

The co-DEGs were imported into the Coremine Medical database (http://www.coremine.com, accessed on 25 May 2022), the relevant traditional Chinese medicine (TCM) information about the gene was downloaded, and the possible therapeutic Chinese medicines were determined at *p* < 0.05.

### 2.8. Clinical Verification and Correlation Analysis

To verify the correlation between cognitive function and T2DM, our research team used random cluster sampling to investigate the current status of dementia among middle-aged and elderly people in the five pilot communities of Zhuhai. We used the simple Intelligent Mental State Examination Scale (MMSE), Montreal Cognitive Assessment (MoCA) Changsha Version, Hamilton Anxiety Scale (HAMA), Hamilton Depression Scale (HAMD), and activities of daily life (ADL) scale for the elderly. Data analysis was carried out using SPSS (version 26.0, IBM Corporation, Armonk, NY, USA), the means and standard deviations for measurement data, and univariate and multivariate logistic regression models to identify cognitive impairment-related risk factors. Meanwhile, we also used correlation analysis to further explore the relationship between dementia, depression, and diabetes.

### 2.9. Validation of the Expression Levels of Hub Genes by qRT-PCR

#### 2.9.1. Patient Data

Elderly patients older than 60 years old and hospitalized in the geriatric department of the Fifth Affiliated Hospital of Sun Yat-sen University from June 2022 to December 2022 were selected as the research subjects. All patients had to undergo cognitive function assessment (using MMSE, MoCA Changsha Version, HAMA, HAMD, and the ADL scale) and clinical assessment (clear diagnosis of the patient’s disease) before enrollment. After cognitive function assessment and baseline data collection, the subjects were divided into 4 groups: 15 in the normal group, 15 in the MCI group, 15 in the T2DM group, and 15 in the MCI + T2DM group. The selection criteria of the microarray datasets and methods for RNA extraction in the dataset are shown in Supplementary S1. There were no statistical differences in age, gender, marital status, and ethnicity among the four groups (Table 2). There were also no differences in the hypoglycemic drugs administered to the T2DM and T2DM + MCI groups. In addition, we analyzed the glycosylated hemoglobin (HbA1c) in the T2DM and T2DM + MCI groups. The results showed that the HbA1c in T2DM patients with mild cognitive impairment was higher than in T2DM patients without MCI (*p* < 0.05). This indicates a special relationship between glycemic control and cognitive impairment in patients with T2DM, and the poorer the glycemic control, the more likely they are to suffer from cognitive dysfunction. However, there may be some deviation due to our small sample size, and future experimental verification of larger samples is still needed.

#### 2.9.2. Quantitative Real-Time Polymerase Chain Reaction (qRT-PCR) Detection

**Figure 1:**
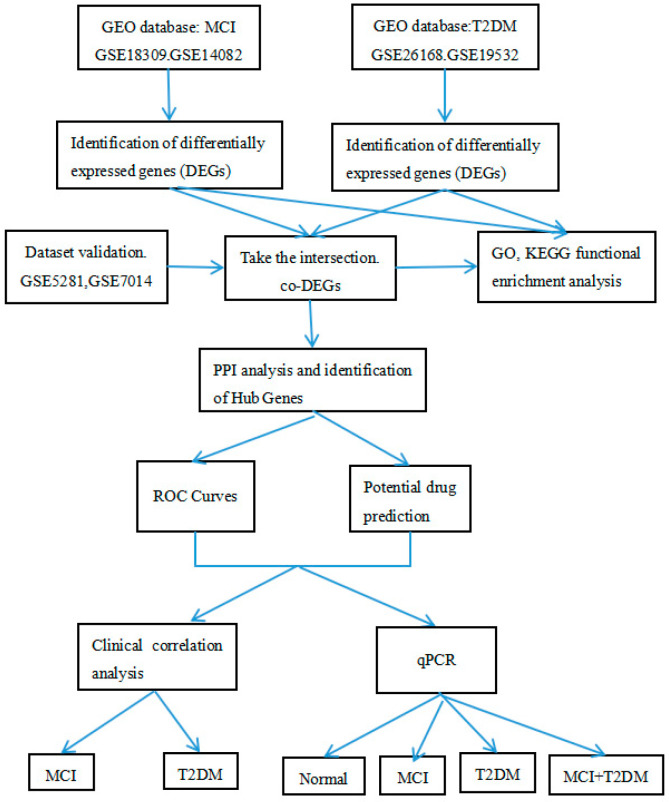
Flowchart for exploring blood co-expressed genes in T2DM and MCI.

In order to further verify the accuracy of the sequencing results of peripheral blood samples in this study, the gold-standard quantitative real-time polymerase chain reaction (qRT-PCR) method was applied for experimental verification of the above-selected key genes to further evaluate their consistency with the sequencing results. Total RNA was extracted from collected serum using the Trizol (Invitrogen, Carlsbad, CA, USA) precipitation method (Takara), and the concentration and purity of RNA were measured using a NanoDrop-2000 spectrophotometer (Thermo Fisher Scientific, Waltham, MA, USA). Subsequently, reverse transcription was performed using HiScript^®^ II Reverse Transcriptase (Vazyme) (Takara Bio Inc., Otsu, Japan), and the operational transcription steps were strictly specified. We followed up on the cDNA obtained for reverse transcription. PCR amplification was carried out using the ChamQ Universal SYBR qPCR Master Mix(Thermo Fisher Scientific, Shanghai, China) kit (Vazyme). The amplification system was as follows: 10 μL of SYBR qPCR mixture, 0.8 μL of primer (IGE Eki Biotechnology, Ltd., Kolkata, India), 0.3 μL of cDNA product, and supplemented with 20 μL of RNase-free water. The PCR reaction conditions were as follows: 95 °C predenaturation for 30 s, 95 °C denaturation for 10 s, and 60 °C annealing for 30 s, for a total of 40 cycles. Dissolution curve: 95 °C for 15 s, 60 °C for 60 s, and 85 °C for 15 s. Three parallel replicate wells were designed in the experiment, and all samples were tested in triplicate. We selected β-actin as the reference gene of this experiment and analyzed the gene expression level using the relative quantification method (2^−ΔΔ^ Ct). The flow chart is presented in Figure 1.

## 3. Results

### 3.1. Identification of Differentially Expressed Genes (DEGs)

**Figure 2:**
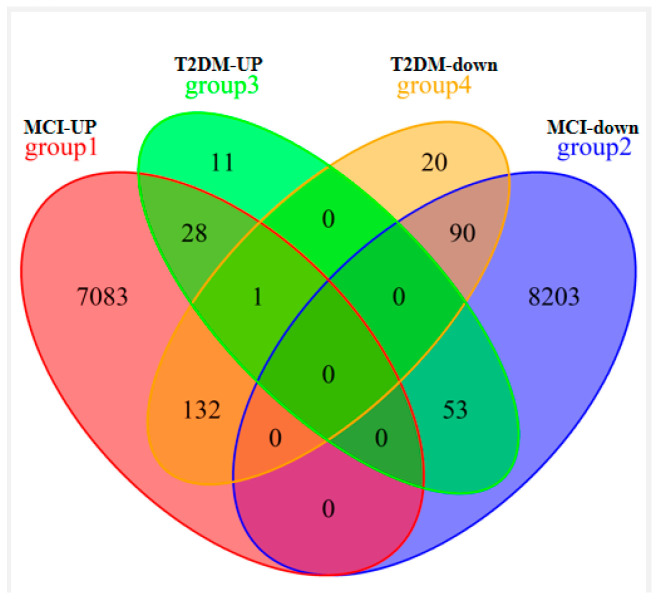
Venn diagram of coDEGs.

A comparison of blood samples from MCI patients with normal controls identified 10,448 DEGs (7083 upregulated genes and 8203 downregulated genes). A comparison of blood samples from T2DM patients and normal controls identified 334 DEGs (92 upregulated genes and 242 downregulated genes). Subsequently, the intersection of MCI-DEGs and T2DM-DEGs was performed on the R platform, their co-DEGs were analyzed, and the results were visualized using a Venn diagram (as seen in Figure 2). We identified 303 co-DEGs, 28 upregulated co-DEGs, and 90 down-regulated co-DEGs. The Supplementary S2 lists all the details of upregulated and down-regulated co-DEGs.

### 3.2. Construction of Co-Expression Modules by WGCNA

**Figure 3:**
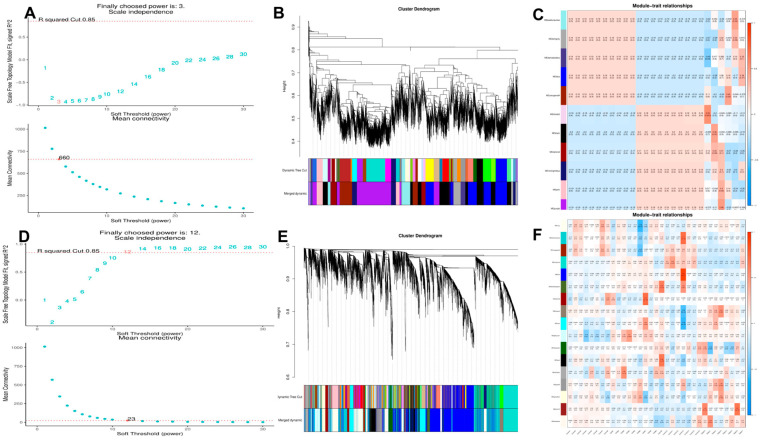
WGCNA for (**A**) the scale-free index for various soft-threshold powers, and (β) the mean connectivity for various soft-threshold powers for MCI. (**B**) Phyloclustering tree map of the genes for MCI. (**C**) Heatmap of module and trait/grouping correlation for MCI. (**D**) The scale-free index for various soft-threshold powers (β) and the mean connectivity for various soft-threshold powers for T2DM. (**E**) Phyloclustering tree map of the genes for T2DM. (**F**) Heatmap of module and trait/grouping correlation for T2DM.

We set the soft threshold to seven to ensure high gene independence and low average connectivity, and then performed the construction of weighted gene co-expression networks to identify co-expressed gene modules. In this study, we analyzed the expression matrices of all of the samples in the MCI and T2DM datasets, respectively, and selected the top 30–50% of the variant genes (less than 5000) for co-expression analysis. The adjacency matrix and the topological overlap matrix were established. We calculated the module signature genes representing the overall gene expression level of each module that were clustered according to their correlation. In the gene co-expression network heatmap, the heatmap regions indicate the non-similarity between the genes, with smaller values and darker colors. In addition, we also draw the correlation of the heat map between a module and a given trait or grouping, the abscissa is the trait or grouping, and the ordinate is the module. The more red the color in the heat map is, the stronger the positive correlation; on the contrary, the more blue the color is, the stronger the negative correlation. The value in the grid is the correlation coefficient and *p*-value, respectively. If a trait or grouping is related to a module closer to the absolute value of 1, then the trait or grouping is most likely related to the gene function of the module. We retained the genes with a correlation in these modules for further analysis. All of the WGCNA analysis results are presented in Figure 3.

### 3.3. Functional Enrichment Analysis

**Figure 4:**
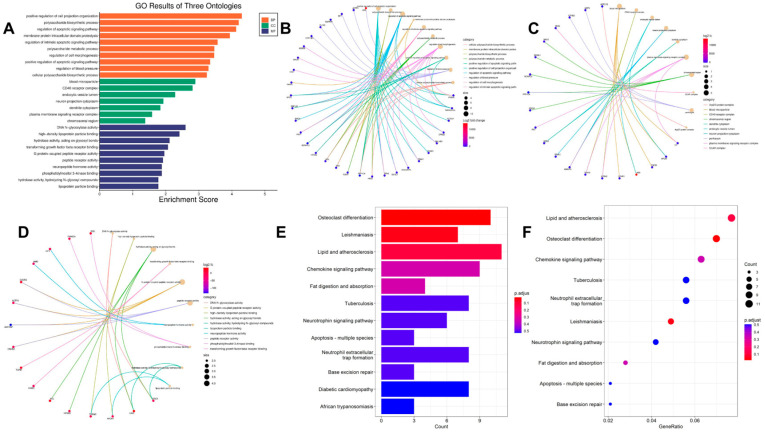
Functional characteristics analysis for the co-DEGs. (**A**) GO enrichment results of three ontologies. (**B**) The BP category of GO enrichment analyses. (**C**) The CC category of GO enrichment analyses. (**D**) The MF category of GO enrichment analyses. (**E**) The barplot of KEGG pathway enrichment results. (**F**) The dotplot of KEGG pathway enrichment results.

We performed GO functional enrichment analysis and KEGG enrichment analysis to further understand the biological function of co-DEGs. The GO enrichment analysis separately identified the biological processes (BP), cellular components (CC), and molecular functions (MF) of the genes. The GO pathway analysis of co-DEGs shows that biological process (BP) changes mainly include glucose metabolism, cell apoptosis, and cell morphology changes. Cell components (CC) are mainly enriched in blood microparticles, CD40 receptor complex, endocytic vesicle lumen, neuron projection cytoplasm, dendrite cytoplasm, plasma membrane signaling receptor complex, and the chromosomal region. In terms of molecular function (MF), co-DEGs are significant in neuropeptide hormone activity, lipoprotein binding, and conversion of growth factor β receptor binding. Figure 4B–D shows the visualization results of the GO enrichment. The results of the KEGG analysis (Figure 4E,F) show that the co-DEGs were mainly enriched in some metabolic-related diseases (fat digestion and absorption (*p*-value = 0.006487), diabetic cardiomyopathy (*p*-value = 0.02558), fat digestion and absorption (*p*-value = 0.00648)), and in the conduction of the signaling pathway (chemokine signaling pathway (*p*-value = 0.006356), AGE-RAGE signaling pathway in diabetic complications (*p*-value = 0.03038), MAPK signaling pathway (*p*-value = 0.0328)).

### 3.4. PPI Network Analysis and Identification of Hub Genes

**Figure 5:**
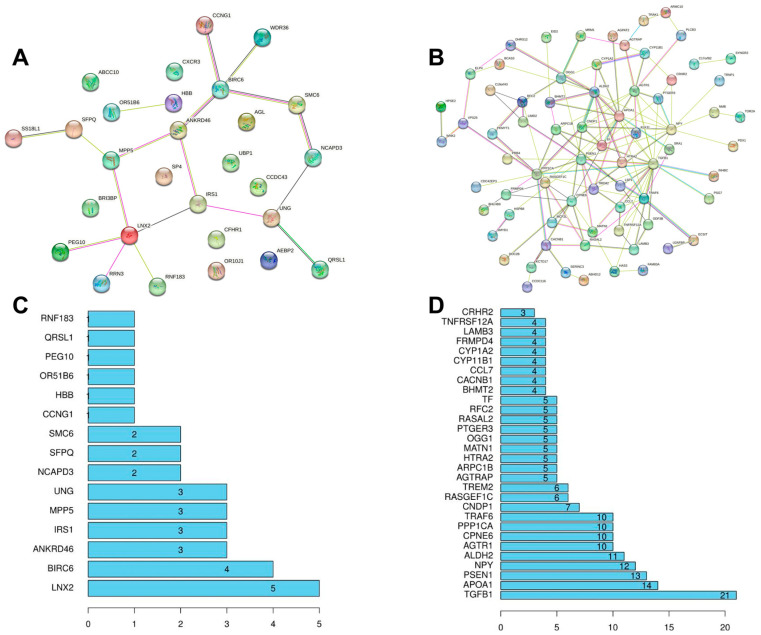
Protein–protein interaction (PPI) analysis of (**A**) upregulated co-DEGs; (**B**) downregulated co-DEGs; (**C**) hub genes in downregulated co-DEGs; (**D**) hub genes in upregulated co-DEGs.

We used STRING databases to construct up-regulated and down-regulated PPI networks to further screen the hub genes of co-DEGs. The up-regulated co-DEGs PPI network consisted of 28 genes, 18 nodes, and 36 edges, while the down-regulated co-DEGs PPI network consisted of 90 genes, 72 nodes, and 282 edges (Figure 5A,B). Meanwhile, the cytoHubba plugin in Cytoscape software was used to select the hub genes. The top 10 differentially expressed genes that were up-regulated were TGFB1, APOA1, PSEN1, NPY, ALDH2, AGTR1, CPNE6, PPP1CA, TRAF6, and CNDP1; while the top 10 that were down-regulated were LNX2, BIRC6, ANKRD46, IRS1, MPP5, UNG, NCAPD3, SFPQ, SMC6, and CCNG1. Finally, nine hub genes of co-DEGs were selected, namely LNX2, BIRC6, ANKRD46, IRS1, TGFB1, APOA1, PSEN1, NPY, and ALDH2 (Figure 5C,D).

### 3.5. Receiver Operating Characteristic Curve Analysis

**Figure 6:**
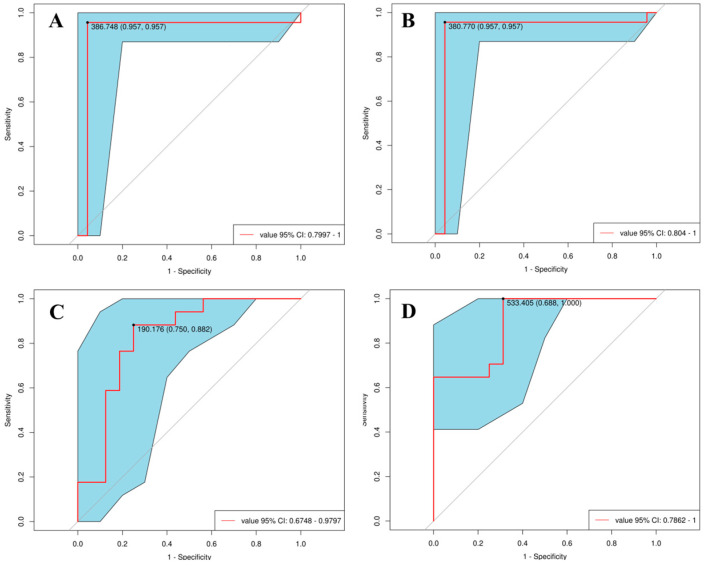
ROC curves of co-DEGs in MCI and T2DM. (**A**) BIRC6 in MCI; (**B**) ALDH2 in MCI; (**C**) BIRC6 in T2DM; (**D**) ALDH2 in T2DM.

We validated the diagnostic value of hub genes in MCI and T2DM using ROC curves. The diagnostic efficiency of the gene for the disease can be assessed based on the AUC values, categorized into low accuracy with an AUC from 0.5 to 0.7, medium accuracy with an AUC from 0.7 to 0.9, and high accuracy if the AUC was above 0.9. As shown in Table 3, the AUC values for both MCI and T2DM showed good diagnostic efficiency (0.9 > AUC > 0.7), with a higher diagnostic value in MCI (AUC > 0.9). Moreover, among the genes with an upregulated expression, BIRC6 has a high diagnostic value for both MCI and T2DM. Among the downregulated genes, ALDH2 had the highest diagnostic value for MCI and T2DM. In the patients with MCI, the AUC of BIRC6 and ALDH2 was 0.9149 (0.7997–1) and 0.9168 (0.804–1), respectively (Figure 6A,B). For T2DM patients, the AUC of BIRC6 and ALDH2 was 0.8272 (0.6748–0.9797), and 0.8934 (0.7862–1), respectively (Figure 6C,D). The ROC curves for all hub genes are shown in Supplementary Figure S2.

### 3.6. Prediction of Potential Drugs and Traditional Chinese Medicine

Upregulated hub genes (LNX2, BIRC6, ANKRD46, and IRS1) and downregulated hub genes (TGFB1, APOA1, PSEN1, NPY, and ALDH2) were submitted to the L1000 platform. Ranked by CMap connectivity score, the top ones indicated similar expression, and the lower ones revealed expression antagonism. These antagonistic small molecules showing antagonism could be drug candidates for treatment. The top 10 and bottom 10 compounds are listed in Table 4.

The nine hub genes were predicted, and Grapsidae, Nymphaea tetragona Georgi, Asini Corii Colla, Angelicae Sinensis Radix, Rosa roxburghii, Arnebia euchroma (Royle) Johnst, Flos leonuri, Periostracum Serpentis, Semen Euryales, Coptis chinensis Franch, Folium Mori, and other traditional Chinese medicines were obtained. Details are given in Table 5. Most of these traditional Chinese medicines have the effect of promoting blood circulation, removing blood stasis, and exerting antioxidant effects [26,27] Combined with the literature reports, we found that these predicted Chinese medicines could achieve therapeutic effects by promoting blood circulation [28]. Many steroid compounds, such as triterpenoids, steroids, and saponins, have been found in these herbs that promote blood circulation. These steroid-like compounds may inhibit Na/K-ATPase, thus achieving the purpose of promoting blood circulation. In addition, the combination of astragalus membranaceus and salviorrhiza can affect the proliferation of NRK-52E cells in a high-glucose environment to promote blood circulation and eliminate blood stasis [29]. Polysaccharides, one of the practical components of traditional Chinese medicine, are found in Coptis chinensis Franch, Allium macrostemon Bunge, and Crataegi Fructus, which have antioxidant, antiviral, and immune-enhancing activities. It has been found that polysaccharides in many plants and microorganisms have significant antioxidant effects, which regulate the expression of downstream antioxidant enzymes mainly through endogenous antioxidant stress Nrf2/ARE pathway [30]. These antioxidant enzymes can further block free radical chain reactions, reducing free radical production. Secondly, inhibiting the expression of iNOS mRNA and reducing NO production can significantly improve the antioxidant capacity and reduce oxidative stress damage [30].

### 3.7. Clinical Verification and Correlation Analysis

A total of 1158 elderly patients aged over 60 years old were screened, and 1094 valid questionnaires were collected, while 64 invalid questionnaires were also collected, of which 11 were age-inconsistent and 53 had incomplete information. The prevalence of dementia in elderly people over 60 years old was 24.63%, the prevalence of depression was 61.57%, and that of anxiety and depression was 43.28%. The prevalence of mild cognitive impairment (MCI) was 23.3%. MCI is an irreversible transitional stage between dementia and normal cognitive performance. It is the best period for people to prevent and intervene in dementia, and a key period for cognitive function exercise. Identifying this stage can effectively reduce the prevalence of senile dementia.

To further explore the risk factors affecting dementia, a univariate logistic regression analysis was performed on the variables that may influence dementia disease in older adults. The results showed that 11 factors were significantly associated with dementia disease (*p* < 0.05): age (OR = 1.085, 95% CI: 1.068–1.101); the literacy level, categorized into the illiterate group, college-level education group or above (OR = 0.135, 95% CI: 0.078–0.233); residence, which was categorized into living alone or living in a non-solitary environment (OR = 0.585, 95%CI: 0.433–0.789); hypertension (OR = 2.826, 95%CI: 2.202–3.627); coronary heart disease (CHD) (OR = 1.742, 95%CI: 1.149–2.642); T2DM (OR = 2.463, 95%CI: 1.740–3.485); cerebrovascular disease (CVD) (OR = 7.396, 95%CI: 4.744–11.530); anxiety disorders (OR = 6.852, 95%CI: 3.839–12.229); and depression (OR = 4.705, 95%CI: 3.173–6.977). Advanced age, living alone, hypertension, diabetes, coronary heart disease, cerebrovascular disease, anxiety, and depression may be risk factors for dementia. A high education level, social living, digestive diseases, kidney disease, and arthritis may be protective factors. Table 6 for details.

To further analyze the risk factors for cognitive impairment, we included 549 people with cognitive impairment as the case group and 545 people without cognitive impairment as the control group, introducing all of the above univariate variables with differential significance for multivariate logistic regression analysis. The results showed that advanced age, living alone, illiteracy, hypertension, diabetes, cerebrovascular disease, anxiety, and depression were the risk factors for dementia in old age. Consistent with our credit analysis, those with a history of T2DM had two times the risk of developing dementia (OR = 1.839, 95%CI: 1.215–2.783) (*p* = 0.004), and those with depression had three times the risk of developing dementia (OR = 3.049, 95%CI: 1.447–6.428) (*p* = 0.003). The details are shown in Table 7.

To investigate the interrelationship between diabetes, depression, and dementia, we performed a correlation analysis. Pearson correlation analysis showed that cognitive impairment and dementia were negatively associated with MoCA scores (r = −0.694, −0.831, *p* < 0.001). Hence, we used the MoCA score to reflect cognitive status. The MoCA score was negatively associated with diabetes and depression scores (r = −0.172, −0.433, *p* < 0.001); cognitive impairment and dementia were positively associated with diabetes and depression scores. Thus, it is preliminarily clear that diabetes and depression affect the occurrence and development of dementia to a certain extent. Details are shown in Table 8.

### 3.8. The qRT-PCR Validation of the Differentially Expressed Genes in the Clinical Samples

**Figure 7:**
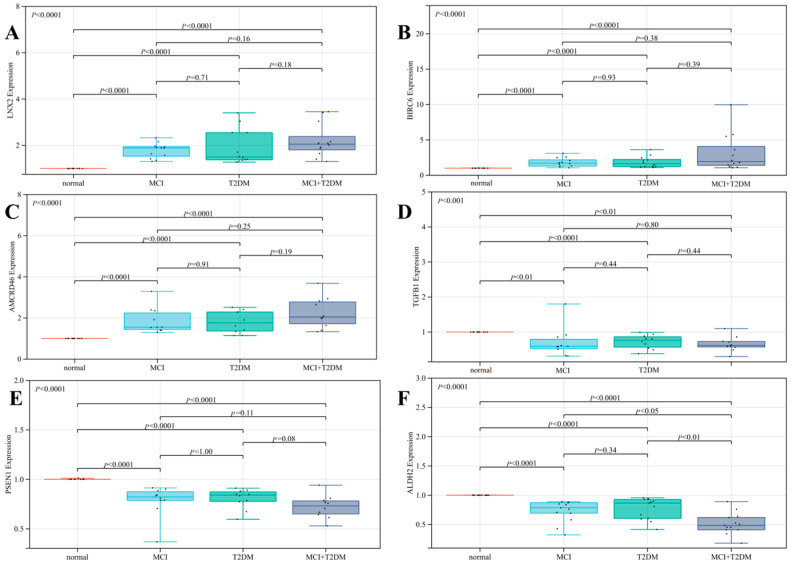
Results of qRT-PCR analysis. (**A**) LNX2; (**B**) BIRC6; (**C**) ANKRD46; (**D**) TGFB1; (**E**) PSEN1; (**F**) ALDH2.

To verify the reliability of the results, qRT-PCR analysis was performed on the four clinical samples (normal, MCI, T2DM, MCI + T2DM) to verify the expression levels of these potential diagnostic biomarkers. Consistent with the results of the credit analysis, the expression levels of LNX2 (*p* < 0.0001), BIRC6 (*p* < 0.0001), and ANKRD46 (*p* < 0.0001) were higher in MCI, T2DM, and MCI + T2DM than in the normal group. The expression level of TGFB1(*p* < 0.001), PSEN1 (*p* < 0.001), and ALDH2 (*p* < 0.001) were significantly lower than normal in the MCI, T2DM, and MCI + T2DM groups. However, the expression of NPY, APOA 1, and IRS1 was low in the blood samples of all patients, and there was no significant difference in gene expression between the normal and disease groups (*p* > 0.05). In addition, we also analyzed the gene expression differences between the MCI, T2DM, and MCI + T2DM groups, and the results were not statistically significant (*p* > 0.05). The boxplot results are shown in Figure 7.

**Figure 8:**
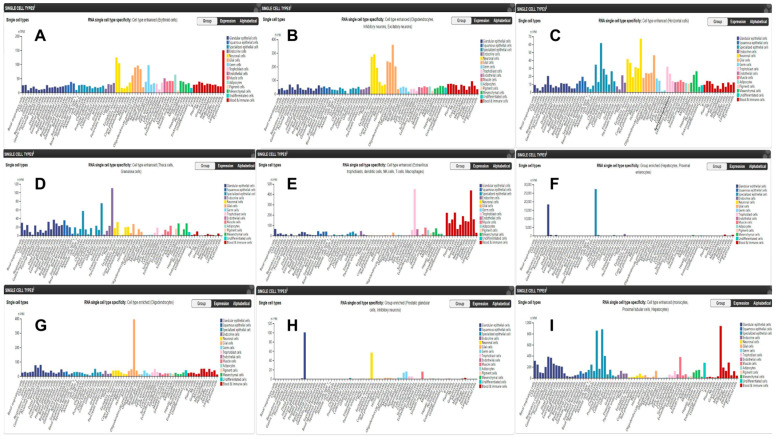
Hub gene expression in human tissues. (**A**) LNX2; (**B**) BIRC6; (**C**) ANKRD46; (**D**) IRS1; (**E**) TGFB1; (**F**) APOA1; (**G**) PSEN1; (**H**) NPY; (**I**) ALDH2.

In addition, we also used the Human Protein Atlas online website (https://www.proteinatlas.org, accessed on 13 May 2022) to verify the expression of nine hub genes in different tissues of the human body (Figure 8). For RNA extraction with reference to the GEO datasets, the hub gene of nucleated cells in the patient’s blood cells was verified by qRT-PCR. According to the literature results (Figure 8), these hub genes are mainly expressed in peripheral monocytes in the blood. Consistent with our experimental results, the expression of NPY, APOA 1, and IRS1 is low in blood samples, so we speculate that these genes are not highly predictive of these diseases. Combined with the above results, LNX2, BIRC6, ANKRD46, TGFB1, PSEN1, and ALDH2 have the potential to be diagnostic biomarkers for MCI and diabetes, although they need to be further validated.

## 4. Discussion

Cognitive disorders have become a common disease in the elderly and are a common cause of disability and death in this age group. The understanding of neurocognitive disorders is still limited, and there are some problems, such as unclear specific mechanisms and the lack of early diagnosis and interventions. Therefore, developing methods for the early identification of and intervention in patients during the MCI period to avoid the development of dementia is crucial. Previous studies have suggested that T2DM is not only a risk factor for MCI, but also promotes the transformation of MCI into dementia [31]. It is widely believed that T2DM and MCI share several common abnormal molecular and cellular characteristics, including impaired glucose metabolism, insulin resistance, and increased oxidative stress, which are manifested by persistent hyperinsulinemia and hyperglycemia [32,33]. Cerebral insulin resistance seems to be an early and common feature in patients with dementia. Abnormal glucose metabolism may play a leading role in the progression of dementia, but its specific mechanism remains unclear. Exploring the common pathogenesis of MCI and T2DM is conducive to the early identification of cognitive impairment to achieve the purpose of diagnosing and treating the disease.

For the first time, we used the blood co-expressed genes of T2DM and MCI as the entry point, performed bioinformatics analysis based on GEO public clinical database, and identified 303 co-expressed genes, 28 up-regulated co-expressed genes, and 90 down-regulated co-expressed genes. We then constructed a KEGG pathway enrichment analysis and PPI network to identify the hub genes in co-DEGs. KEGG pathway analysis of co-DEGs is mainly concentrated in several metabolic diseases and signaling pathways. However, surprisingly, the pathway enrichment analysis, which was mainly concentrated on neurodegenerative diseases, was not statistically significant (*p*-value > 0.05). Our analysis is mainly due to the low number of co-DEGs, but this does not mean that these co-expressed genes are not associated with neurodegenerative diseases. From the above analysis, we identified the hub genes of the nine co-DEGs, namely LNX 2, BIRC 6, ANKRD46, IRS 1, TGFB1, APOA 1, PSEN 1, NPY, and ALDH 2. The ROC curve analysis found that these nine hub genes had excellent diagnostic values for both MCI and T2DM (0.9 > AUC > 0.7). Based on the results of bioinformatics analysis, on the one hand, we designed a clinical verification to explore the risk factors and correlation analysis of cognitive impairment. The current investigation results showed that MCI was correlated with T2DM, a risk factor for cognitive impairment. The risk of MCI in patients with a history of T2DM was 1.839 times higher than that in patients without diabetes. On the other hand, we designed a qRT-PCR experiment to detect the relative expression of nine hub genes in the blood tissues of the normal group, the MCI group, the T2DM group, and the MCI + T2DM group, respectively. The experimental results showed that the expression trends of LNX2, BIRC6, ANKRD46, PSEN1, and ALDH2 were consistent with our bioinformatics results, while there was no significant difference in the expression of NPY, APOA1, and IRS1, which was considered as being due to its low expression in the blood. Combined with the above findings, we found that these five hub genes play an important role in the progression and diagnosis of MCI and T2DM, but their specific mechanisms need to be further studied. In addition, we found that LNX2 and ALDH2 had better results. We guessed that they may act on the same pathway and may have antagonistic effects, so the expression is highly correlated.

### 4.1. Potential Diagnostic Markers

Currently, some dementia-related genes (such as APOE, MPKA, and APP/PS1) have been found to have good clinical diagnostic value. However, most patients undergo genetic testing at an advanced stage of the disease, and it does not prevent the disease from developing. We obtained co-expressed genes of MCI and T2DM through bioinformatics analysis, and we can detect co-expressed genes for early diagnosis of the disease. Combined with bioinformatics analysis and our qRT-PCR validation results, we considered that LNX2, BIRC6, ANKRD46, TGFB1, PSEN1, and ALDH2 could be good disease markers.

LNX2 is mainly expressed in the hepatocytes, as well as erythroid cells, B cells, and T cells in the blood. The LNX2 gene is a member of the LNX (numb protein-X ligand) family and mainly encodes LNX2 proteins. LNX proteins usually contain an amino-terminal RING domain adjacent to two or four PDZ domains, which is unique to the LNX family [34]. The LNX2 gene is expressed in all tissues throughout the body and is strongly expressed in the forebrain and many other regions of the developing embryo. Contact protein-associated protein-4 (CASPR4) is a transmembrane protein that is a member of the neuropathic superfamily. It is expressed in the developing cortex and neural progenitor cells in the SVZ. Some studies have shown that LNX2 binds to the cytoplasmic domain of CASPR4, and both proteins have been found to inhibit proliferation and promote differentiation in cultured neural progenitor cells [35]. Currently, LNX2 has not been used in studies related to cognitive impairment and T2DM.

The BIRC6 gene is widely expressed in oligodendrocytes, inhibitory neurons, and excitatory neurons, and also in erythroid cells (dendritic cells, B cells, T cells, and erythroid cells). BIRC6 encodes proteins with the BIR (baculovirus inhibiting apoptosis protein repeat) 999 domain and the UBCc (ubiquitin coupling enzyme E2) domain. This protein inhibits apoptosis by promoting ubiquitination to degrade apoptotic proteins. At present, most people are familiar with the BIRC6 gene in tumors, such as non-small cell lung cancer, bladder cancer, colorectal cancer, and others [36,37,38]. Ubiquitin coupling enzyme (BRUCE) containing BIR repeats of the apoptotic protein family (IAP) has been reported to inhibit the regulation of autophagosome and lysosome fusion, and also interacts with syntaxin 17 (STX17), an important mediator of autophagosome–lysosome fusion. BRUCE may affect the development of dystrophic axons in Alzheimer’s disease by regulating the fusion of autophagosomes and lysosomes, thus affecting cognition [39]. In addition, it has been reported that BIRC6 may be a potential biomarker of T2DM, but its mechanism of action still needs further study [40].

ANKRD46 was mainly enriched in hepatocytes and thyroid gland cells and encodes proteins containing multiple ankyrin repeats. The ankyrin domain plays a role in protein–protein interactions in various cellular processes. Alternative splicing results in multiple transcript variants. Through the literature search, we found that there were few studies on the ANKRD46 gene, which mainly plays a pathogenic role in tumors, such as nasopharyngeal carcinoma, breast cancer, and gastric cancer [41].

TGFB1 (TGF-β1) is widely expressed in extravillous trophoblasts, NK cells, dendritic cells, and T cells in humans. The TGFB1 gene encodes a secreted ligand of the transforming growth factor-β (TGF-β) protein superfamily. Ligands of this family bind to various TGF-β receptors, leading to the recruitment and activation of SMAD family transcription factors that regulate gene expression. The TGFB1 gene encodes proteins that regulate cell proliferation, differentiation, and growth, as well as the expression and activation of other growth factors (such as interferon-gamma and tumor necrosis factor α) [42]. T2DM leads to increased TGFβ signaling, which interferes with VEGFA-induced monocyte migration and leads to monocyte dysfunction. TGFβ signaling can affect monocyte function, causing vascular complications [43] in T2DM patients. Studies have found that serum TGF-β1 levels in patients with Alzheimer’s disease (AD), vascular dementia (VaD), and Parkinson’s disease dementia (PDD) are significantly increased, but serum TGF-β1 levels in AD and VaD are significantly higher than those in PDD [44]. TGF-β1 may be a biomarker for assessing the degree of cognitive impairment [44].

The PSEN 1 gene (PS1) is mainly expressed in oligodendrocytes and encodes for presenilin 1. Most patients with familial hereditary AD carry mutations in the PSEN gene. These disease-associated mutations can lead to increased production of Aβ. Presenolin can regulate APP processing through γ-secretase. Current studies have shown that PSEN1 is mainly associated with Alzheimer’s disease, amyloidosis, cancer-related genes, cardiomyopathy, disease variation, and cognitive disorders [45,46]. PSEN1 is a well-known gene associated with MCI and AD [47]. In addition, studies have found that PSEN1 is also associated with T2DM, and this gene promotes Aβ deposition, which is a factor in the development of diabetes to AD [48].

The ALDH2 gene is mainly expressed in hepatocytes, and the proximal renal tubular cells, and it is also expressed in blood cells. The ALDH2 gene encodes for the aldehyde dehydrogenase 2 family member, a protein that belongs to the proteins of the aldehyde dehydrogenase family [49]. Aldehyde dehydrogenase is the second enzyme in the main oxidative pathway of alcohol metabolism. Increased exposure to acetaldehyde in individuals with catalytically active forms may also lead to greater susceptibility to many types of cancer [50]. Some research teams have reported that patients with T2DM and mutations in the formaldehyde (FA)-degrading enzyme aldehyde dehydrogenase 2 (ALDH2) gene have higher levels of FA and more severe dementia [51]. Overexpression of ALDH2 reduces FA and reduces hyperglycemia and cognitive deficits in a diabetic mouse model [52].

In conclusion, combined with the results of the literature search and bioinformatic analysis, we believe that the three genes, BIRC6, TGFB1, and PSEN1, have a clear correlation with T2DM and cognitive impairment, which can be used as target genes for disease prediction in the future. At the same time, more experiments can be designed around these genes to verify the pathogenic mechanism, in order to achieve the purpose of early disease prediction.

### 4.2. Potential Treatment Options

Although the treatment of dementia has been studied for many years, only two classes of drugs are currently approved for the treatment of AD, namely cholinesterase inhibitors and N-methyl-D-aspartic acid antagonists (NMDA). Although both drugs are clinically effective, they can only improve symptoms, not cure or prevent the disease [53,54]. At present, the treatment of MCI mainly focuses on non-pharmaceutical therapy, such as cognitive function training and physical therapy, and there is a lack of specific drug therapies [55]. Therefore, exploring drugs that enable the early treatment of MCI is particularly important for human social health.

As Table 3 shows, the lower the CMap connectivity score, the more likely it is to be a potential therapeutic agent. Mycophenol ester is an antimetabolite and potent immunosuppressant used as an adjunct therapy for the prevention of allograft rejection and the treatment of severe autoimmune diseases. It was found that mycophenolate mofetil could be used to treat T2DM and diabetic ketoacidosis and improve insulin resistance [56]. In addition, some researchers have designed animal experiments to show that mantemycophenol ester has therapeutic effects in diabetic rat models, and has a certain protective effect on experimental diabetic nephropathy. At present, few researchers use mycophenolate mofetil to treat MCI, but it can be used to treat neuropsychiatric symptoms caused by systemic erythema [57]. Nilotinib, a serine/threonine kinase mammalian sterile 20-like kinase 1 (MST1) inhibitor, is widely used for adjunctive treatment of early breast cancer. MST1 can directly induce β-cell death and impair insulin secretion. The importance of MST1 as a therapeutic target for diabetes has been demonstrated at the β-cell level and in diabetic complications [58]. Neratinib is a potential β cytoprotective drug, and both in vivo and in vitro experiments have demonstrated that neratinib can treat T1DM and T2DM [59]. In addition, neratinib can also activate the ataxic telangiectasia mutation (ATM) through reactive oxygen species, which can phosphorylate and activate the AMP-dependent protein kinase (AMPK). By reducing mTOR activity and directly activating ULK1, AMPK also leads to the formation of autophagosomes and the degradation of Tau and APP, thus achieving the purpose of treating dementia. However, no studies have observed the therapeutic effects of Merck60 and benzanthrone in diseases, and more studies may be needed in the future.

At the same time, we have obtained co-expressed important pathogenic genes and identified targeted Chinese medicines according to the target genes. In this paper, hub genes were used to screen traditional Chinese medicines with potential therapeutic effects, such as Grapsidae, Nymphaea tetragona Georgi, Asini Corii Colla, Angelicae Sinensis Radix, Rosa roxburghii, Arnebia euchr0ma (Royle) Johnst, Flos leonuri, Periostracum Serpentis, Semen Euryales, Coptis chinensis Franch, and Folium Mori. Compared with the different concepts of Western medicine, traditional Chinese medicine believes that the etiology and pathology of dementia are complex and diverse, which can be divided into a deficiency of qi and blood, a functional decline of the five viscera, six organs, blood stasis, phlegm turbidity, and blockage [60,61]. Some studies have found that bupleurum can significantly inhibit Aβ-induced cell death, increase cell membrane potential and reduce mitochondria-dependent apoptosis induced by Aβ by up-regulating the ratio of Bcl-2/Bax, thus achieving a therapeutic effect on AD [62]. In addition, some research teams have found that Bupleurum Shugan pills can have a therapeutic effect on diabetic patients with depression by effectively reducing the HbA1c level and depressive symptom score, improving clinical efficacy, and regulating serum 5-HT and NE levels, and are safe and reliable. Bupleurum Shugan pills are a traditional Chinese medicine prescription, and their main components are bupleurum, green peel, tangerine peel, parsnip, Fructus aurantii, Fructus aurantii, Radix xylosa, and Radix Aconitum. Based on UHPLC-Q-Exactive-ObitriapMS, researchers quickly analyzed the chemical components of Bupleurum Shugan pills [63]. A total of 121 compounds were detected, including flavonoids, terpenoids, phenylpropanes, anthraquinones, and alkaloids. Modern pharmacological studies have shown that flavonoids have the effects of lowering blood sugar, regulating mood, and anti-depression. In addition, it has been proved that puerarin can inhibit tau phosphorylation in the olfactory bulb of AD rats, and its mechanism may be due to its decreased activity level of GSK-3β and its effect on glucose metabolism in diabetic model rats [63]. Other traditional Chinese medicines, such as Asini Corii Colla, angelica, coptis, and so on, have a good effect on the treatment of cognition-related diseases and diabetes [64,65,66]. These results all indicate that the prediction results of traditional Chinese medicine in this study are consistent with clinical practice. However, the specific mechanisms are still unclear and need to be further explored. However, this is a meaningful research direction.

We expect that exploring the co-expressed genes of MCI and T2DM can clearly determine the co-pathogenesis and further serve as new therapeutic targets for the diagnosis and treatment of diseases. However, some limitations still exist in this study. First, when we selected the target dataset in the GEO database, we could only select from a limited number of datasets, as some dataset sample sizes were too small, while some datasets had no obvious differentially expressed genes (adj *p*-value < 0.05, and |logFC| ≥ 1.0), such as GSE48350, GSE131617, GSE15993, and GSE161335. We performed a multi-chip co-analysis of the same disease and standardized the dataset to eliminate differences across platforms and individuals. Through the verification of the dataset, we found that our conclusion is still reliable. In addition, the GEO database is still being updated, as are disease-causing genes, and there are still more genes to be discovered. During clinical verification, due to the impact of the COVID-19 pandemic, our team only analyzed the current situation regarding cognitive impairment in Zhuhai City, lacking multicenter verification. Meanwhile, since January 2022, Zhuhai has been affected by the COVID-19 pandemic, hindering the progress of the project, and the sample size is limited. In addition, one of the randomly selected communities was a nursing home, where most of the elderly people have a cognitive impairment, which makes the data biased to a certain extent. In the future, large-sample, multicenter experiments are needed to study the correlation between MCI and T2DM. In the experimental part of qPCR, we expected to include more clinical samples to verify our results, but due to the strict inclusion and exclusion criteria we set, the number of cases meeting the criteria was limited, but we tried to include as many clinical samples as possible to verify our conclusions. In addition, we obtained only a preliminary conclusion, and further verification of cell and animal models needs to be performed. In the future, we will conduct further research around our conclusions.

## 5. Conclusions

We used a series of bioinformatics methods to screen and validate the hub genes common to MCI and T2DM. MCI and T2DM have blood co-expression genes, and we identified the key pathogenic genes, namely LNX2, BIRC6, ANKRD46, IRS1, TGFB1, APOA1, PSEN1, NPY, and ALDH2, which all have good predictive value. At the clinical level, through a current situation investigation, we confirmed the correlation between MCI and T2DM, which is a risk factor for cognitive impairment, and they share a common pathogenesis. LNX2 and ALDH2 may be potential genetic markers and new targets for the diagnosis and treatment of MCI and T2DM, which are promising for delaying the occurrence and development of dementia. The results of this study may further suggest that T2DM patients are prone to complications relating to cognitive impairment and have a tendency to develop AD, and can further predict the early development of AD, thus providing a reference for the treatment of T2DM and dementia. However, future cell and animal studies are needed to explore the mechanisms that may influence the development and progression of gene-regulated diseases.

## Figures and Tables

**Table 1:** Datasets details.

Diseases	GEO ID	Platform	Organism	Type	Samples (Case vs. Control)	Ethnicity/Race	PMID
MCI	GSE18309	GPL570	Homo sapiens	Blood	3 vs. 3	Taiwanese	24381135
	GSE140829	GPL15988	Homo sapiens	Blood	20 vs. 20	Caucasian	34225819
	GSE5281	GPL570	Homo sapiens	Hippocampus	13 vs. 10	Caucasian	34367470
T2DM	GSE26168	GPL6883	Homo sapiens	Blood	18 vs. 18	Singaporean	33149760
	GSE15932	GPL570	Homo sapiens	Blood	8 vs. 8	Chinese	34257566
	GSE7014	GPL570	Homo sapiens	Skeletal Muscle	6 vs. 20	Caucasian	31797865

**Table 2:** Patient baseline data.

Group	Normal	MCI	T2DM	MCI + T2DM	*p*
age	62.2667 ± 3.88158	65.0000 ± 5.29150	65.0000 ± 5.94018	65.7333 ± 7.28469	0.371
male	3(20)	6(60)	10(66.7)	5(33.3)	0.65
female	12(80)	9(40)	5(33.3)	10(66.7)	
HBA1C			6.8200 ± 0.81082	8.3813 ± 1.64171	0.03

**Table 3:** Diagnostic value of hub genes in MCI and T2DM.

Gene	MCI-AUC	MCI-95%CI	T2DM-AUC	T2DM-95%CI
LNX2	0.9112	0.796–1	0.8051	0.6454–0.9649
BIRC6	0.9149	0.7997–1	0.8272	0.6748–0.9797
ANKRD46	0.9112	0.796–1	0.8419	0.7049–0.979
IRS1	0.9074	0.7921–1	0.8162	0.6703–0.962
TGFB1	0.8809	0.7515–1	0.8897	0.7686–1
APOA1	0.9168	0.804–1	0.8676	0.7304–1
PSEN1	0.9149	0.7997–1	0.8897	0.7583–1
NPY	0.8771	0.7438–1	0.864	0.7318–0.9961
ALDH2	0.9168	0.804–1	0.8934	0.7862–1

**Table 4:** The top 10 and bottom 10 potential compounds.

Rank	Score	ID	Name	Description
1	98.94	BRD-K95402279	geranylgeraniol	Farnesyltransferase inhibitor
2	98.31	BRD-A35338386	NECA	Adenosine receptor agonist
3	98.2	BRD-K53972329	ruxolitinib	JAK inhibitor
4	97.31	BRD-K11853856	PJ-34	PARP inhibitor
5	97.02	BRD-K50388907	fenofibrate	PPAR receptor agonist
6	96.99	BRD-A53952395	prilocaine	Local anesthetic
7	96.92	BRD-K18855837	varenicline	Acetylcholine receptor agonist
8	95.86	BRD-K86873305	piperacillin	Bacterial cell wall synthesis inhibitor
9	95.66	BRD-K33453211	levocabastine	Histamine receptor antagonist
10	94.11	BRD-K48722833	iloperidone	Dopamine receptor antagonist
11	−92.52	BRD-K50311478	tosyl-phenylalanyl-chloromethyl-ketone	Chymotrypsin inhibitor
12	−93.48	BRD-K72895815	SSR-69071	Leukocyte elastase inhibitor
13	−93.62	BRD-K25504083	cytochalasin-d	Actin polymerization inhibitor
14	−94.04	BRD-A02481876	importazole	Importin-beta transport receptor inhibitor
15	−94.05	BRD-A52650764	ingenol	PKC activator
16	−94.75	BRD-K83837640	JNJ-26854165	HDAC inhibitor
17	−95.35	BRD-K85606544	neratinib	EGFR inhibitor
18	−95.84	BRD-K92428153	mycophenolate-mofetil	Dehydrogenase inhibitor
19	−97.74	BRD-K04466929	Merck60	HDAC inhibitor
20	−99.23	BRD-K76872913	benzanthrone	Aromatic hydrocarbon derivative

**Table 5:** Potential traditional Chinese medicines.

Gene	Traditional Chinese Medicine
LNX2	Grapsidae
BIRC6	-
ANKRD46	-
IRS1	Nymphaea tetragona Georgi, Semen Euryales, Coptis chinensis Franch, Folium Mori
TGFB1	Asini Corii Colla, Angelicae Sinensis Radix, Rosa roxburghii, Arnebia euchr0ma(Royle) Johnst, Flos leonuri
APOA1	Allium macrostemon Bunge, Crataegi Fructus, Carthamus tinctorius, AchilleaptarmicaL, Celastrus orbiculatus Thunb, Terminalia catappa L, chao xian yin yang huo
PSEN1	Paris polyphylla, Corylus chinensis, Fructus Perillae Frutescentis, Radix Perillae, Caulis Perillae Furtescentis, Liquidambar species, Herba Dendrobii, Brassica chinensis L, Fructus Toosendan, Calyx Kaki
NPY	Radix et Rhizonma Ephedrae, Ephedra intermedin Schrenk, Colla Carapacis Trionycis, Caput Trionycis, Trionycis Carapax, Carpesium abrotanoides, Osmanthus fragrans
ALDH2	Hovenia dulcis thunnb, Pueraria lobata, Pueraria montana, Radix Oryzae Glutinosae, Artemisia capillaris, Bupleurum chinense, Semen Oryzae Sativae

**Table 6:** Univariate logistic regression analysis of the risk factors for senile dementia.

	B	S.E.	Wald	df	Sig.	Exp(B)	95% C.I. for EXP(B) Lower Upper	
Gender	−0.005	0.122	0.002	1	0.965	0.995	0.783	1.264
Age	0.081	0.008	105.862	1	0.000	1.085	1.068	1.101
Married	−1.190	0.285	17.495	1	0.000	0.304	0.174	0.531
Divorced	−0.950	0.423	5.055	1	0.025	0.387	0.169	0.885
Widowed	−0.455	0.316	2.072	1	0.150	0.635	0.342	1.179
Living situation	−0.537	0.153	12.342	1	0.000	0.585	0.433	0.789
Education			87.923	4	0.000			
Primary school	−0.872	0.250	12.118	1	0.000	0.418	0.256	0.683
Junior middle school	−1.670	0.258	42.004	1	0.000	0.188	0.114	0.312
Senior middle school	−1.827	0.267	46.742	1	0.000	0.161	0.095	0.272
College degree or above	−2.001	0.278	51.626	1	0.000	0.135	0.078	0.233
Hypertension	1.039	0.127	66.662	1	0.000	2.826	2.202	3.627
CHD	0.555	0.212	6.841	1	0.009	1.742	1.149	2.642
T2DM	0.901	0.177	25.851	1	0.000	2.463	1.740	3.485
CVD	2.001	0.227	77.986	1	0.000	7.396	4.744	11.530
Kidney disease	0.333	0.589	0.320	1	0.572	1.395	0.440	4.422
Digestive disease	−0.467	0.293	2.533	1	0.111	0.627	0.353	1.114
Hyperlipidemia	−0.080	0.219	0.133	1	0.715	0.923	0.601	1.418
Arthritis	0.010	0.190	0.003	1	0.959	1.010	0.696	1.466
HAMA	1.549	0.201	59.356	1	0.000	4.705	3.173	6.977
HAMD	1.924	0.296	42.392	1	0.000	6.852	3.839	12.229

**Table 7:** Multivariate logistic regression analysis of the risk factors for senile dementia.

	B	S.E.	Wald	df	Sig.	Exp(B)	95% C.I. for EXP(B) Lower Upper	
Age	0.059	0.009	40.179	1	0.000	1.061	1.041	1.080
Living situation	−0.531	0.186	8.129	1	0.004	0.588	0.408	0.847
Education			69.658	4	0.000			
Primary school	−0.617	0.195	10.049	1	0.002	0.540	0.369	0.790
Junior middle school	−0.822	0.211	15.145	1	0.000	0.439	0.290	0.665
Senior middle school	−1.364	0.234	33.934	1	0.000	0.256	0.162	0.405
Illiteracy	0.905	0.279	10.562	1	0.001	2.472	1.432	4.268
Hypertension	0.694	0.155	19.984	1	0.000	2.003	1.477	2.715
CHD	−0.155	0.257	0.366	1	0.545	0.856	0.518	1.416
T2DM	0.609	0.211	8.296	1	0.004	1.839	1.215	2.783
CVD	1.647	0.248	44.052	1	0.000	5.193	3.193	8.446
HAMA	0.946	0.264	12.812	1	0.000	2.574	1.534	4.320
HAMD	1.115	0.380	8.586	1	0.003	3.049	1.447	6.428
Constant	−4.166	0.691	36.375	1	0.000	0.016		

**Table 8:** Pearson correlation analysis.

		Diabetes	HAMD	MOCA	Cognitive Disorder	Dementia
Diabetes	Pearson Correlation	1	0.142	−0.172	0.157	0.131
	Sig. (2-tailed)		0.000	0.000	0.000	0.000
	N	1094	1094	1094	1094	1094
HAMD	Pearson Correlation	0.142	1	−0.433	0.400	0.399
	Sig. (2-tailed)	0.000		0.000	0.000	0.000
	N	1094	1094	1094	1094	1094
MOCA	Pearson Correlation	−0.172	−0.433	1	−0.694	−0.831
	Sig. (2-tailed)	0.000	0.000		0.000	0.000
	N	1094	1094	1094	1094	1094
Cognitive disorder	Pearson Correlation	0.157	0.400	−0.694	1	0.600
	Sig. (2-tailed)	0.000	0.000	0.000		0.000
	N	1094	1094	1094	1094	1094
Dementia	Pearson Correlation	0.131	0.399	−0.831	0.600	1
	Sig. (2-tailed)	0.000	0.000	0.000	0.000	
	N	1094	1094	1094	1094	1094

Correlation is significant at the 0.01 level (2-tailed).

## Data Availability

The datasets used and analyzed during the current study are available from the corresponding author on reasonable request.

## Supplementary Materials

The following supporting information can be downloaded at: https://www.mdpi.com/article/10.3390/biomedicines11040993/s1, Figure S1: Volcano maps of (A) AD; (B) T2DM; Figure S2: ROC curve of co-DEGs in MCI, T2DM; Table S1: Primers used for quantitative PCR; Supplementary S1: Supplementary Note of Materials and Methods; Supplementary S2. upregulated and down-regulated co-DEGs.

## Abbreviations

MCI, mild cognitive impairment; AD, Alzheimer’s disease; T2DM, type 2 diabetes mellitus; qRT-PCR, quantitative real-time polymerase chain reaction; ATP, adenosine triphosphate; AGE, advanced terminal glycation; ROS, reactive oxygen species; NFTs, neurofibrillary tangles; Aβ, amyloid β-protein; GEO, Gene Expression Omnibus; HbA1c, glycosylated hemoglobin; WGCNA, weighted gene co-expression network analysis; DEGs, differentially expressed genes; KEGG, Kyoto Encyclopedia of Genes and Genomes; GO, Gene Ontology; BP, biological processes; CC, cellular component; MF, molecular function (MF); co-DEGs differentially co-expressed genes; PPI, protein–protein interaction; ROC, receiver operating characteristic curve analysis; AUC, area under curve; TCM, traditional Chinese medicine; MMSE, Intelligent Mental State Examination Scale; MoCA, Montreal Cognitive Assessment; HAMA, Hamilton Anxiety Scale; HAMD, Hamilton Depression Scale; ADL, activities of daily life; OR, odds ratio; CI, confidence interval; CHD, coronary heart disease; CVD, cerebrovascular disease; APP, Alzheimer precursor protein; FA, formaldehyde; NMDA, N-methyl-D-aspartic acid antagonists; MST1, mammalian sterile 20-like kinase 1; ATM, ataxic telangiectasia mutation.
