# Supplementary material for: Exploration and Clinical Verification of the Blood Co-Expression Genes of Type 2 Diabetes Mellitus and Mild Cognitive Dysfunction in the Elderly
Source: Biomedicines. 2023 Mar 23;11(4):993. doi: 10.3390/biomedicines11040993 (PMC10135937; doi:10.3390/biomedicines11040993)

Supplementary S1. Supplementary Note of Materials and Methods

2.1 Acquisition MCI-related and T2DM-related gene expression data

The selection criteria of microarray datasets were as follows: 1. It met the diagnostic criteria of MCI and T2DM, and one data set included the diseased and the normal control groups; 2. The patient is older than 60 years old; 3. Considering that blood samples are more readily available than other tissue samples in clinical work, sample type we chose as the blood sample; 4. The research target of the data set was total RNA; 5. The validation datasets were derived from the same platform, and the patient's gender and race were consistent. All the extracted molecules that we included in the datasets were total RNA. Total RNA was extracted using Trizol reagent (Invitrogen). Total RNA from nucleated blood cells was following lysis of erythrocytes and removal of cell debris.

2.2 Construction of weighted gene co-expression network analysis (WGCNA)

Weighted gene co-expression network analysis (WGCNA) is a common method to construct gene co-expression networks. The WGCNA analysis method aims to find the jointly expressed gene modules (module), and to explore the association relationship between the gene networks and the phenotypes of interest, the core genes in the network, as well as the enrichment analysis for each module gene. WGCNA is divided into two parts: expression quantity cluster analysis and phenotype association, which mainly includes gene co-expression network construction, module identification, module information extraction, module and trait association, and the regulatory relationship of genes within modules.

2.6 Conduction of Receiver Operating Characteristic (ROC) Curves

Receiver Operating Characteristic Curve Analysis (ROC) curves were mainly used for the prediction accuracy of the X-axis on the Y-axis. Initially, the ROC curve was used in the military, but now more often in the medical field, to determine whether a particular factor is of diagnostic value for diagnosing a specific disease. The ROC plots are the curves reflecting the relationship between sensitivity and specificity. The Area Under Curve (AUC) is the area under the ROC curve with values between 0.5 and 1. The closer the AUC is to 1.0, the higher the authenticity of the detection method, which is equal to 0.5, then the authenticity is the lowest, and there is no application value.

2.9 Validation of the expression levels of hub genes by qRT-PCR

2.9.1 Patient Data

This study was approved by the Medical Ethics Committee of our hospital. Inclusion criteria included meeting the diagnostic criteria for mild cognitive dysfunction and the diagnostic criteria for T2DM proposed in the China Clinical Guidelines for the Prevention and Treatment of T2DM (2020 edition). Exclusion criteria include patients with previous mental disorders such as major depression, schizophrenia and delusion, cognitive disorders caused by material poisoning and systemic diseases, patients with the acute phase of major diseases such as acute and critical diseases, and patients with malignant tumors; patients with recent chronic inflammation, metabolic diseases (Type 1 diabetes, latent autoimmune diabetes in adults, diabetic ketoacidosis, hyperosmolarity syndrome, hyperuricemia, severe obesity, thyroid disease), acute diseases, colds and infectious diseases. The patients and their families gave informed consent to the experiment, and they all signed the informed consent form.

Supplementary S2. upregulated and down-regulated co-DEGs

Gene ID	Gene symbol	Gene name	Type
900	CCNG1	cyclin G1	up
89845	ABCC10	ATP binding cassette subfamily C member 10	up
23310	NCAPD3	non-SMC condensin II complex subunit D3	up
57448	BIRC6	baculoviral IAP repeat containing 6	up
3043	HBB	hemoglobin subunit beta	up
7374	UNG	uracil DNA glycosylase	up
3078	CFHR1	complement factor H related 1	up
26039	SS18L1	SS18L1subunit of BAF chromatin remodeling complex	up
7342	UBP1	upstream binding protein 1	up
134430	WDR36	WD repeat domain 36	up
121536	AEBP2	AE binding protein 2	up
853734	RRN3	rDNA-binding RNA polymerase I transcriptional factor	up
851099	SMC6	DNA repair protein SMC6	up
26476	OR10J1	olfactory receptor family 10 subfamily J member 1	up
55278	QRSL1	glutaminyl-tRNA amidotransferase subunit QRSL1	up
124808	CCDC43	coiled-coil domain containing 43	up
222484	LNX2	ligand of numb-protein X 2	up
140707	BRI3BP	BRI3 binding protein	up
157567	ANKRD46	ankyrin repeat domain 46	up
138065	RNF183	ring finger protein 183	up
109448382	MPP5	membrane palmitoylated protein 5	up
390058	OR51B6	olfactory receptor family 51 subfamily B member 6	up
23089	PEG10	paternally expressed 10	up
2833	CXCR3	C-X-C motif chemokine receptor 3	up
3667	IRS1	insulin receptor substrate 1	up
6671	SP4	Sp4transcription factor	up
6421	SFPQ	splicing factor proline and glutamine rich	up
178	AGL	amylo-alpha-1, 6-glucosidase,<br>4-alpha-glucanotransferase	up
6945	MLX	MAX dimerization protein MLX	down
27429	HTRA2	HtrA serine peptidase 2	down
19164	PSEN1	presenilin 1	down
4828	NMB	neuromedin B	down
7307	U2AF1	U2 small nuclear RNA auxiliary factor 1	down
6707	SPRR3	small proline rich protein 3	down
853566	VPS25	ESCRT-II subunit protein VPS25	down
107057624	C19orf43	chromosome 19 open reading frame 43	down
6452	SH3BP2	SH3 domain binding protein 2	down
9144	SYNGR2	synaptogyrin 2	down
10955	SERINC3	serine incorporator 3	down
51295	ECSIT	ECSITsignaling integrator	down

12922	CRHR2	corticotropin releasing hormone receptor 2	down
23263	MCF2L	MCF.2 cell line derived transforming sequence like	down
1584	CYP11B1	cytochrome P450 family 11 subfamily B member 1	down
26090	ABHD12	abhydrolase domain containing 12, lysophospholipase	down
83787	ARMC10	armadillo repeat containing 10	down
285596	FAM153A	family with sequence similarity 153 member A	down
13447	DOC2B	double C2, beta	down
5982	RFC2	replication factor C subunit 2	down
3038	HAS3	hyaluronan synthase 3	down
27433	TOR2A	torsin family 2 member A	down
6354	CCL7	C-C motif chemokine ligand 7	down
4146	MATN1	matrilin 1	down
10011	SRA1	steroid receptor RNA activator 1	down
12295	CACNB1	calcium channel, voltage-dependent, beta 1 subunit	down
51330	TNFRSF12A	TNF receptor superfamily member 12A	down
84985	FAM83A	family with sequence similarity 83 member A	down
14536	NR6A1	nuclear receptor subfamily 6, group A, member 1	down
148066	ZNRF4	zinc and ring finger 4	down
335524	ODF3B	outer dense fiber of sperm tails 3B	down
3914	LAMB3	laminin subunit beta 3	down
18828	PLSCR2	phospholipid scramblase 2	down
11012	KLK11	kallikrein related peptidase 11	down
91977	MYOZ3	myozenin 3	down
26353	HSPB8	heat shock protein family B (small) member 8	down
79734	KCTD17	potassium channel tetramerization domain containing 17	down
10555	AGPAT2	1-acylglycerol-3-phosphate O-acyltransferase 2	down
23743	BHMT2	betaine--homocysteine S-methyltransferase	down
7189	TRAF6	TNF receptor associated factor 6	down
9362	CPNE6	copine 6	down
5545	PRB4	proline rich protein BstNI subfamily 4	down
1544	CYP1A2	cytochrome P450 family 1 subfamily A member 2	down
163126	EID2	EP300 interacting inhibitor of differentiation 2	down
7018	TF	transferrin	down
12180	SMYD1	SET and MYND domain containing 1	down
217	ALDH2	aldehyde dehydrogenase 2 family member	down
18609	PDX1	pancreatic and duodenal homeobox 1	down
26610	ELP4	elongator acetyltransferase complex subunit 4	down
69539	TRNP1	TMF1-regulated nuclear protein 1	down
5331	PLCB3	phospholipase C beta 3	down
11610	AGTRAP	angiotensin II, type I receptor-associated protein	down
80774	LIMD2	LIM domain containing 2	down
57474	ZNF490	zinc finger protein 490	down
9758	FRMPD4	FERM and PDZ domain containing 4	down
70237	BHLHB9	basic helix-loop-helix domain containing, class B9	down

388407	C17orf82	long intergenic non-protein coding RNA 2875	down
3626	INHBC	inhibin subunit beta C	down
335	APOA1	apolipoprotein A1	down
5499	PPP1CA	protein phosphatase 1 catalytic subunit alpha	down
54828	BCAS3	BCAS3microtubule associated cell migration factor	down
283579	C14orf178	chromosome 14 open reading frame 178	down
11945	ATP4B	ATPase, H+/K+ exchanging, beta polypeptide	down
245	ALOX12P2	arachidonate 12-lipoxygenase pseudogene 2	down
79946	C10orf95	chromosome 10 open reading frame 95	down
91661	ZNF765	zinc finger protein 765	down
4852	NPY	neuropeptide Y	down
854376	MRM1	Mrm1p	down
4968	OGG1	8-oxoguanine DNA glycosylase	down
65268	WNK2	WNK lysine deficient protein kinase 2	down
164592	CCDC116	coiled-coil domain containing 116	down
10095	ARPC1B	actin related protein 2/3 complex subunit 1B	down
54209	TREM2	triggering receptor expressed on myeloid cells 2	down
7040	TGFB1	transforming growth factor beta 1	down
185	AGTR1	angiotensin II receptor type 1	down
84167	C19orf44	chromosome 19 open reading frame 44	down
9462	RASAL2	RAS protein activator like 2	down
401565	FAM166A	family with sequence similarity 166 member A	down
60495	HPSE2	heparanase 2 (inactive)	down
22905	TRAK1	trafficking kinesin protein 1	down
9088	PKMYT1	protein kinase, membrane associated tyrosine/threonine 1	down
237987	OTOP2	otopetrin 2	down
255426	RASGEF1C	RasGEF domain family member 1C	down
284379	LOC284379	solute carrier family 7 member 3 pseudogene	down
19218	PTGER3	prostaglandin E receptor 3 (subtype EP3)	down
84735	CNDP1	carnosine dipeptidase 1	down
5676	PSG7	pregnancy specific beta-1-glycoprotein 7	down
4046	LSP1	lymphocyte specific protein 1	down
10602	CDC42EP3	CDC42 effector protein 3	down
79758	DHRS12	dehydrogenase/reductase 12	down

Table S1. Primers used for quantitative PCR

gene	Primer sequences
LNX2	Forward primer: CCCAGCCTGGTAACACCATT
	Reverse primer: AACGGTCATGCCAAGGGATT
BIRC6	Forward primer: CTGTGAGTTCCTTCGGGGTT
	Reverse primer: TCTCTTGCAGCACCTGTAGC

ANKRD4	Forward primer: CGTTCGAGTCGCAGATCCTT
6	Reverse primer: TGGGTCAAAGCCACTTTCCA
IRS1	Forward primer: AGAGGACCGTCAGTAGCTCA
	Reverse primer: ACTGAAATGGATGCATCGTACC
TGFB1	Forward primer: GGAAATTGAGGGCTTTCGCC
	Reverse primer: GAGGTCCTTGCGGAAGTCAA
APOA1	Forward primer: AGAGACTGCGAGAAGGAGGT
	Reverse primer: TCTCTGCCGCTGTCTTTGAG
PSEN1	Forward primer: GGGAAGCGTATACCTAATCTGGGAG
	Reverse primer: ACGTACAGTATTGCTCAGGTGG
NPY	Forward primer: CGCTGCGACACTACATCAAC
	Reverse primer: AGGCCAGAGAGCAAGTCTCA
ALDH2	Forward primer: GGAAGATGTGGACAAGGCAGT
	Reverse primer: GGGATGGTTTTCCCGTGGTA

Figure S1. Volcano maps of (A) AD; (B) T2DM

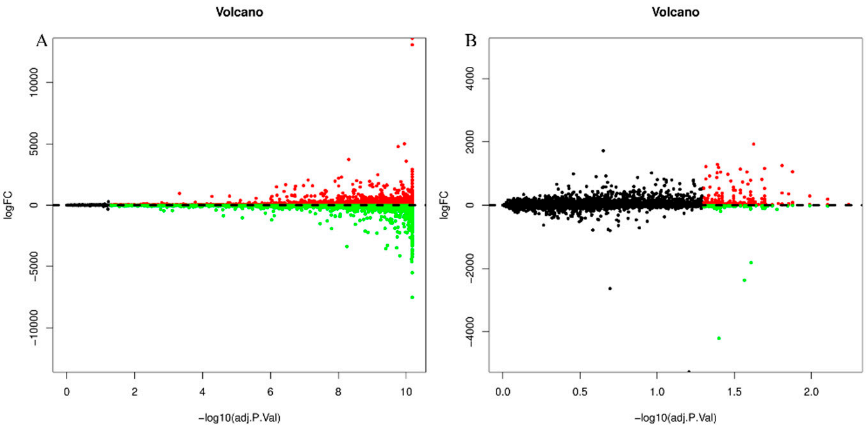

Figure S2. ROC curve of co-DEGs in MCI, T2DM. (A) LNX2 in MCI; (B) BIRC6 in MCI; (C) ANKRD46 in MCI; (D) IRS1 in MCI; (E) TGFB1 in MCI; (F) APOA1 in MCI; (G) PSEN1 in MCI; (H) NPY in MCI; (I) ALDH2 in MCI; (J) LNX2 in T2DM; (K) BIRC6 in T2DM; (L) ANKRD46 in T2DM; (M) IRS1 in T2DM; (N) TGFB1 in T2DM; (O) APOA1 in T2DM; (P) PSEN1 in T2DM; (Q) NPY in T2DM; (R) ALDH2 in T2DM.

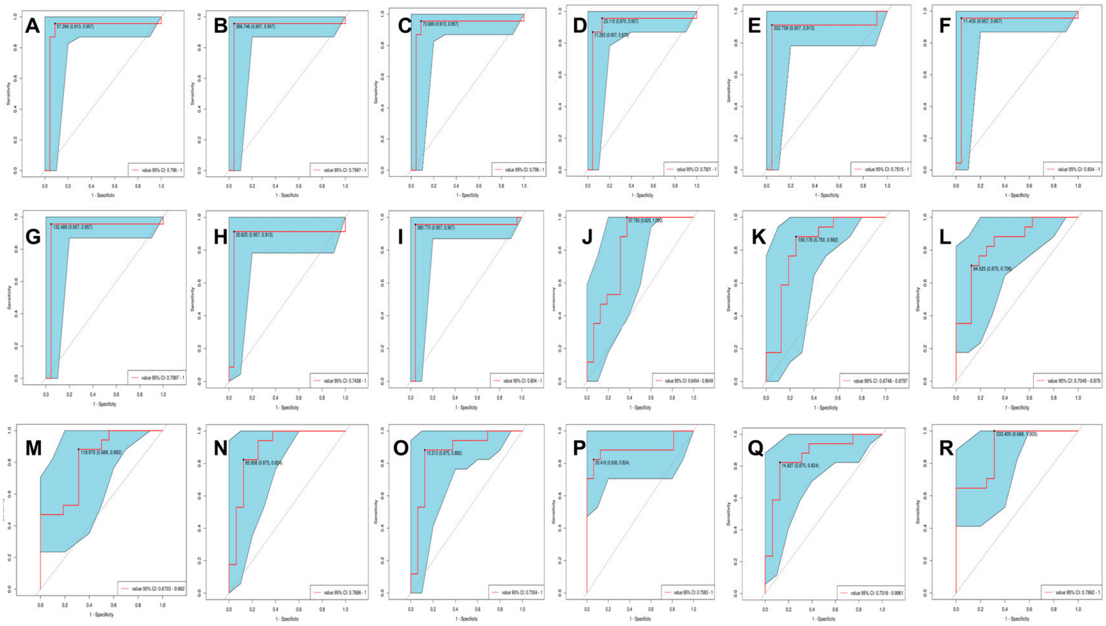

Supplement: Supplementary file 1 [file biomedicines-11-00993-s001.zip › biomedicines-2137479-supplementary.pdf]
